# Interpretation of Hormone Levels in Older Patients: Points for Consideration

**DOI:** 10.1155/2012/712425

**Published:** 2012-05-14

**Authors:** Krystyna Sztefko, Patrycja Szybowska

**Affiliations:** Clinical Biochemistry Department, College of Medicine, Jagiellonian University, Wielicka Street 265, 30-663 Kracow, Poland

## Abstract

Blood hormone and tumor marker concentrations are usually determined by immunochemical methods which are based on an unique reaction between antigen and assay capture antibody. Despite the speed and simplicity of assays performance on automatic immunochemistry platforms, the interpretation of final results requires a deep knowledge of method fallibility. General lack of immunoassays standardization, presence of cross-reacting substances in patient's sample, limitation of free hormones measurement due to abnormal analyte binding protein concentrations, assay interferences due to patient's autoantibodies, and heterophilic antibodies, as well as proper interpretation of very low- and very high-sample analyte levels, are the main points discussed in respect to hormones and tumor markers measurement in geriatric population.

## 1. Introduction

Aging process is associated with physiological changes in function of almost every organ and system, including the endocrine system. The function of endocrine glands function declines progressively with age. For example, dehydroepiandrosterone sulphate (DHEAS) concentration is about 10–20% of maximum in patients at the age of 70–80 years [1]. The activity of the growth hormone/IGF axis also declines steadily, and a sudden cessation of the function of some elements of the hormonal system is well documented [2–5]. Negative feedback loop is the common regulatory mechanism within endocrine system. Thus, markedly decreased or markedly increased blood hormones concentrations can be measured in elderly patients. Multiple diseases, which frequently entail polytherapy with the use of multiple drugs, influence the levels of hormones *in vivo *and the measurement biomarkers as well as drugs concentration by immunochemistry *in vitro *[6]. The coexistence of multiple diseases may cause secondary changes in hormone levels as it is the case with the level of thyroid-stimulating hormone (TSH) in nonthyroidal diseases [7–9]. Moreover, in an elderly population, the presence of autoantibodies due to autoimmune or chronic diseases is more frequent than it is in the younger population. The presence of autoantibodies in the plasma or serum, like rheumatoid factors (RFs), may affect the determination of many hormones by immunochemistry, or if directed against a specific molecule, such as prolactin or troponin, they may mislead the medical decision based on hormone measurement [10–13]. In addition, changes in the serum level of specific and nonspecific hormone-binding protein, especially albumin, frequently observed in geriatric population, greatly influence the measurement of free hormones [14]. Furthermore, the fallibility and limitation of immunochemical methods of hormone measurements may lead to clinically misleading interpretation of laboratory results of hormone concentration in elderly population.

Hormones, proteins, peptides, tumor markers, and drugs are routinely measured using automated immunochemistry platforms. Immunochemistry methods are based on the reaction between an antigen and an antibody; both competitive and noncompetitive method formats are used. The reaction between antigen and antibody is very specific due to unique properties and stereochemistry of epitope on the antigen and paratope on the antibody. Although the analytical procedure for measuring hormones is very simple and easy to perform, the interpretation of results requires not only medical knowledge but also deep knowledge of immunochemistry limitations. This is especially true when hormone measurements are performed in the serum of the patient at an extremely advanced age, the patient with multiple or chronic disease, and the patient on multiple drugs therapy.

For all the laboratory determinations, the preanalytical phase of a diagnostic procedure contributes the most to the total laboratory error of measurement, regardless of the patient's age. The type of anticoagulants used, the presence of hemolysis, lipemia, hyperbilirubinemia, and paraproteinemia are well-known factors, influencing the measurement of biochemical markers, including hormones. The observed bias due to hemolysis may be negative, as it is the case with cortisol, parathyroid hormone (PTH), and insulin measurement, or positive as it is the case with troponin I determination [15, 16]; or susceptibility to interference by hemolysis is different, as it was shown for cardiac troponin I and troponin T measured by current immunoassays [17]. Some hormones are very labile *ex vivo,* and negative bias due to proteolysis is frequently seen in the measurement of peptides, such as adrenocorticotropic hormones (ACTH), insulin, osteocalcin, C-peptide, and PTH [18, 19]. Although the concentrations of most hormones are measured directly in the serum or plasma, it is necessary for many hormones to treat the blood sample specifically before analysis, as in the case of gastrointestinal peptides measurement [20, 21].

Apart from the aforementioned pre non-specific analytical problems, there are many pitfalls which may occur during the analytical phase of hormones determination by immunochemical methods, which are known by the laboratory personnel but frequently unknown by physicians. For a proper interpretation of the hormone concentration results, the comparison of the results with appropriate reference intervals coupled with good clinical knowledge is necessary. In case of discrepancy between the laboratory data and the clinical picture of the patient, repeated analytical measurements are usually requested. However, in the case of hormones and tumor markers, repeated measurements of the analyte by immunochemistry in questionable patients' samples give concentration results that do not always meet clinical expectations. To avoid such a situation, it is important for clinicians to know and to understand the limitation and fallibility of immunochemical methods in order to protect the patient from misdiagnosis. This is extremely important for every patient, but it must be stressed that in samples from geriatric patients, the presence of various drugs and their metabolites, the presence of autoantibodies and other inducible antibodies, and low albumin level, as well as disturbances in specific and nonspecific hormone-binding protein levels, are frequently observed. In addition, tumor marker measurements are much more frequently requested in older patients as compared to other age groups, extremely high level of some proteins can be expected as well. On the other hand, after surgery of the endocrine gland due to cancer, or during suppressive therapy, the measurement of very low level of some hormones is important for clinical management of a geriatric patient. Thus, for proper interpretation of the laboratory results of hormones and tumor markers determination, it is advisable for physicians to become familiar with most important immunochemistry issues, so that they could answer the following questions: (a) what is being measured by a given immunoassay? (b) how accurate are low and high concentrations of hormone/tumor marker measured? (c) how do binding proteins affect hormone measurement? (d) how do autoantibodies; heterophilic and anti-animal antibodies interfere with the measurement of hormone/tumor marker?

### 1.1. What Is Being Measured by the Immunoassay?

Different chemical molecules, such as protein, peptides, biogenic amines, steroids, and drugs, can be measured by immunochemical methods. As for any other methods, standardization of immunochemical methods is necessary to ensure accuracy of a measurement and comparability of results between different assays. However, most of the immunoassays lack proper standardization. Although the primary standards are available for small molecules (amines, steroids, and drugs), the lack of commutability between primary or secondary standards and the patient's samples due to matrix effect make the standardization process a very difficult task. On the other hand, many hormones of clinical interest are present in the blood in heterogeneous forms (growth hormone, prolactin, gonadotropins, TSH, and gastrin) [22–25] or in monomeric and dimeric forms (insulin) [26, 27]. For heterogeneous molecules, the exact definitions of the substance intended to be measured by immunoassay should always be specified by manufacturers because depending on the specificity of antibodies used in immunochemical methods, different forms of protein can be measured [28]. In addition, plasma samples contain a vast variety of molecules, and there is always a possibility that a chemical structure recognized by an immunoassay capture antibody can be found not only on the molecule of interest, but also on “cross-reacting” substances. Heterogeneity of proteins and their structural similarity and the presence of cross-reacting substances in patients' samples can be a source of false positive (competitive methods) or false negative (noncompetitive methods) results [28–31]. In case of heterogenous proteins, harmonization can be used to improve comparability of methods. The purpose of the harmonization process is to obtain similar results for the analyte measured by different “harmonized” immunoassays calibrated with the use of the same calibrator. It has to be stressed out that harmonization of methods is not equivalent to method standardization.

Immunochemical methods for the measurement of the same analyte may differ with respect to reagent antibodies and to a different standard for calibration. As a consequence, the results of the concentration of hormones and tumor markers obtained by different assay or immunochemistry platforms are often not comparable. Thus, two issues are of great importance: firstly, the knowledge of the molecule that is being measured by immunoassay; secondly, the mandatory use of the method-dependent reference intervals established by the laboratory. Taking into account the lack of immunoassays standardization, heterogeneity of many peptides and protein, structural similarities of steroids and their metabolites, as well as capture antibody specificity, the request for the hormone or tumor marker measurement by two laboratories using different immunoassays should be avoided.

### 1.2. How Accurate Are Low and High Concentrations of the Hormone/Tumor Marker Being Measured?

Analytical sensitivity is an important issue for those analytes for which low concentrations in the patient's sample are diagnostically important as it can be observed in geriatric population in case of C-reactive protein (CRP), estrogen, TSH, and troponin measurement [32–35]. Interpretation of very low concentrations of some analytes requires understanding the analytical sensitivity which, in the simplest way, can be defined as the lowest hormone concentration that can be determined by a given method. From the clinical point of view, it is not enough to accept the limit of the absence of the analyte in the sample based on a repeated zero standard measurement and taking the mean value plus two or three standard deviations. Since each analytical measurement is burdened with error, it is important to know what is the limit of analyte quantification (functional sensitivity of the method). For proper interpretation of a low analyte level, it is necessary to know the concentration value that is measured with certain, predefined precision, usually 10–20%. Many methods are not sensitive enough to measure, for example, low estrogen level (characteristic for pediatric population, men and postmenopausal women) or TSH in geriatric population with nonthyroidal illnesses. It should be noted that a laboratory cannot determine the concentration of any analyte below the functional sensitivity with acceptable precision and accuracy, and any approximation of standard curve below the value of concentration determining functional sensitivity is the unacceptable analytical practice. It is especially important when C-reactive protein (CRP) is not measured by highly sensitive CRP method (hsCRP), and clinical judgment is made on the basis of the CRP level as a prognostic factor in a geriatric patient. The measurement of CRP by two immunoassays differing in analytical sensitivity and using the results interchangeably, that is, both as an inflammatory marker and a prognostic marker of future cardiovascular events should never be done. Another example showing the importance of functional sensitivity in geriatric population is troponin measurement, since a very small increase in its concentration may have serious clinical consequences [36]. Each laboratory that measures troponin level should have functional sensitivity established under routine conditions, and physicians should be aware of such concentration value in order to avoid patient misdiagnosis.

Measurement of very high or extremely high concentration of hormones and tumor markers is a great challenge for laboratory staff, since disagreement between the clinical picture of the patient and the laboratory result is sometimes noted. This is especially true for a geriatric population because the frequency of oncologic diseases of different origin increases with age. In immunochemical noncompetitive methods, unlike in other analytical methods, a high-dose effect (hook effect) may occur. In such methods, antigen is linked with two assay antibodies (solid-phase capture antibody and signal antibody) forming a so-called “sandwich”, and the proportionality between the assay signal and analyte concentration is seen. However, the enormous amount of the analyte in the patient's sample blocks both assay capture and labeled antibodies, which does not allow for the formation of a typical “sandwich” [28]; and the linear relationship between the magnitude of the assay signal and the concentration of analyte no longer exists. As a consequence, assay signal is descending. This means that the same assay signal is obtained for the low and the very high analyte concentration. As a result, falsely low, frequently normal, concentration of the analyte is measured [37]. This effect occurs more frequently in homogenous noncompetitive assays compared to heterogeneous noncompetitive assays. If hook effect is suspected, each laboratory performs the dilution test until stable assay signal is obtained. Erroneous results due to hook effect can be observed, among others, for carcinoembryonic antigen (CEA), alpha-fetoprotein (AFP), prostate-specific antigen (PSA), prolactin, thyroglobulin, and cancer-antigen CA-125 [38–44]. Requesting the tumor marker measurement, either for the patient diagnosis or for monitoring therapy, and suspecting the hook effect in an oncologic patient are of great importance because only the abnormal concentration results (outside reference intervals) alert laboratory personnel and physicians; the results within normal range or below cutoff points rarely undergo additional laboratory procedure, unless there is a disagreement between the laboratory result and the patient clinical condition.

### 1.3. How Do Binding Proteins Affect Hormone Measurement?

In the geriatric population, the results of hormones measurement should be interpreted with caution as age-related decline in concentration is characteristic not only for specific binding proteins, such as insulin-like growth factor-binding protein 3 (IGFBP-3), but also for nonspecific binding proteins, such as albumin [45–47]. It may be expected that decreased serum albumin concentration is related to aging; however, it is caused more often by chronic malnutrition than by aging itself [48]. Prolonged decrease in albumin concentration is characteristic of liver and kidney disease, congestive heart disease, and protein-losing enteropathies. Inflammation, frequently seen in elderly population, is another cause of low albumin level, as albumin is a negative acute-phase protein [49]. Changes in specific levels of hormone-binding proteins influence the discrete equilibrium which exists between bound and free hormone fraction. Changes in nonspecific binding protein concentration do not only influence the balance between free and bound fraction of hormone but also have a great impact on the plasma level of many biochemical parameters as these proteins bind and release different ligands, changing the sample matrix. This is especially true for albumin, which is an universal carrier for drugs, metals, fatty acids, vitamins, steroids, minerals, and hormones; binding/releasing different ligands strongly depends on pathological condition [50–53].

A good example of the effect of binding proteins on hormone determination is the estimation of free-thyroid hormone levels (FT4 and FT3) in elderly population. The FT4 plasma concentration depends on the binding capacity of thyroxine-binding globulin concentration (TBG) as well as albumin and prealbumin. Depending on the immunoassay format, either false positive (competitive methods) or false negative (noncompetitive methods) results of free hormone measurement can be obtained as an effect of binding proteins interference [28]. Interpretation of FT4 is also difficult in a female patient on estrogen therapy because estrogen excess is associated with a rise of TBG concentration as well as in a patient on androgen therapy in whom a marked decreased TBG level is observed [54].

In geriatric patients treated with heparin (including low-molecular-weight heparin), a misleading diagnosis can affect the patients' safety due to falsely elevated FT4. The concentration of FT4 in such patients depends on the time that elapsed between heparin administration and blood sampling as well as the time that elapsed between the collection of the blood and performing immunoassay measurement [55]. Free fatty acids released due to *in vitro* lipolysis displace T4 from its binding protein complexes. Thus, a false increase in FT4 concentration is seen. In relation to this, in the interpretation of hormone level in the plasma in both free and bound fraction, a low albumin level should always be taken into consideration.

Lower serum albumin level frequently observed in geriatric population is also associated with the decrease in maximum binding capacity of drugs, which is significant during polytherapy. As a consequence, free-drug concentrations in the plasma are increased [56]. Common drugs used in the geriatric population strongly bind to plasma protein (tricyclic antidepressants, psychotropic medications, benzodiazepines, phenytoin and warfarin). Hence, any disturbance in binding proteins influences the plasma drug concentration measured by immunochemistry.

### 1.4. How Do Autoantibodies, Heterophilic, and Anti-Animal Antibodies Interfere with Hormone/Tumor Markers Measurement?

Common health problems encountered in the geriatric population include various chronic inflammatory diseases such as rheumatoid arthritis, pneumonia, and systemic lupus erythematosus (SLE) [57–60]. In such conditions, the presence of different autoantibodies in the blood is observed, and the prevalence of increased concentration of autoantibodies increases with age [61, 62]. Changes in the immune system are associated with a high incidence of antibodies such as rheumatoid factors (RFs), antinuclear antibodies (ANA), antibodies against single-strand DNA (ssDNA), antibodies against double-strand DNA (dsDNA), antiphospholipid antibodies, antibodies against hormones (i.e., antibodies against insulin), and anty-IGFBP2 antibodies [61]. In plasma samples, they represent a large variation in titer at different periods of time, depending on the patient's disease stage. In the geriatric population, the presence of organ and nonorgan specific autoantibodies is common; and frequently, no visible symptoms of disease are seen [61]. Autoimmune thyroid disease is a frequent pathology in geriatric patients which includes Graves' disease, Hashimoto's thyroiditis, hyperthyroidism, and hypothyroidism. Therefore, increased levels of autoantibodies against thyroglobulin (antyTg), autoantibodies against thyrotropin receptor (TRAB), and antithyroid peroxidase antibodies (anti-TPO) are also noted [63].

From the analytical point of view, different problems have to be considered if it is necessary to measure the concentration of protein against which autoantibodies are present in the plasma sample or measurement of autoantibodies as an independent marker of immune disease is requested. Firstly, autoantibodies can interfere with the analyte measurement, giving erroneous results and, depending on the assay format, both underestimation (noncompetitive methods) or overestimation (competitive methods) can be observed [28]. The most typical example is the thyroglobulin concentration measurement in the presence of antithyroglobulin antibodies in a patient with differentiated thyroid carcinoma [64–67]. Secondly, if the level of autoantibodies is to be measured, final results depend, to a large extent, on the amount of endogenous self-antigen already bound to autoantibodies and frequently, no agreement between competitive and noncompetitive immunoassay formats is achieved. Thirdly, human immunoglobulin frequently forms a complex with the target protein intended to be measured, forming a so-called macroprotein. The complex of prolactin with immunoglobulin is one of the well-recognized protein macroform by endocrinologists. A complex of monomeric protein with immunoglobulin, usually IgG, may not be active *in vivo* but can be immunoreactive, so it can be detected by immunoassays [28]. It is assumed that macroprotein has no biological activity, but its half-life is much longer than the half-life of monomeric protein. Thus, macroprotein accumulates in the blood. Depending on the immunoassay format, both free and complexed forms of protein may be detected by the immunoassay or only the free form is measured but with macroprotein invisible by the assay antibody. Recently, analytical problems connecting the measurement of troponin in a sample in the presence of autoantibodies against troponin T and troponin I have been discussed in the literature [68–70]. Since the frequency of protein macroform formation increases with age, it is important to take such interference into consideration in case of disagreement between clinical picture of patient and laboratory immunoassay result.

In addition to autoantibodies against self-antigens, any inducible antibodies against different foreign antigens can be present in human serum samples. Such inducible antibodies are usually polyreactive and are directed against poorly defined foreign antigens. For analytical purposes they have been called heterophilic antibodies. The best known heterophilic antibodies are rheumatoid factors [71]. In serum or plasma samples, human anti-animal antibodies produced against antigen of animal origin, such as human anti-mouse antibodies (HAMA) or human anti-rabbit antibodies (HARA), can also be present [72–74]. Both human heterophilic and human anti-animal antibodies have properties similar to autoantibodies with respect to binding to immunoassay reagent antibodies [75]. This means that these human antibodies have the ability to interfere in an immunoassay reaction by binding to reagent assay antibodies (animal origin, usually mouse monoclonal antibody) either by binding to the epitope or by sterically blocking the access of antigen to the binding site. The type, amount, and affinity of human interfering antibodies (heterophilic or anti-animal) in a patient's sample are usually unknown and variable [76–79]. The mechanism of heterophilic antibody interference depends on the assay format; both falsely elevated, falsely positive, and falsely decreased results can be obtained. It is impossible to predict the mode of reaction between assay reagent antibodies and interfering antibodies just as it is almost impossible to judge a priori in which patient's sample interference will occur.

In the geriatric population, interference from heterophilic antibodies is as extremely important as the variety of antibodies that are present in the blood. For example, rheumatoid factors are present in 70% of patients with rheumatoid arthritis and the occurrence of human anti-mouse antibodies as a consequence of treating the patient with mouse immunoglobulin for diagnostic and therapeutic purposes is estimated as high as 11.7% [80]. In the literature, a plethora of papers describe the unexpected interference from anti-animal antibodies, for example, in the PTH measurement in serum sample of a patient treated with murine monoclonal antibody directed toward different surface antigens of human T-cell immunoglobulin [81]. It is expected that the problem of interference from heterophilic and human anti-mouse antibodies will increase as new therapeutic approaches for cancer treatment are introduced.

## 2. Conclusions

Accuracy of analytical measurement of different biochemical parameters is a prerequisite for proper diagnosis and treatment monitoring of the patient. Immunochemical methods play an important role in measurement of a variety of biochemical molecules, although due to their fallibility, many limitations in measurement are noted. Immunochemistry is a very powerful analytical technique, but imperfections in analytical measurement are directly connected with unique basis of methods, general lack of standardization, and presence of many interfering substances in patients' samples. The more a patient's sample matrix differs from the normal sample matrix, the higher the probability that erroneous results will occur. In older patients, misinterpretation of immunochemistry results due to the presence of interfering endogenous substances (cross-reacting substances, abnormal hormone binding proteins, presence of autoantibodies, heterophilic antibodies, and anti-animal antibodies) in the blood is more frequent than in younger individuals. It has to be stressed, that most pitfalls in analyte measurement by immunochemistry are related to a patient's sample, and no quality control assurance program exists to protect patient from erroneous results. The only way to suspect an error in immunochemistry results is through the information obtained from physicians, where there is disagreement between laboratory results and the patient's condition. Each laboratory has procedures to look for errors in immunochemistry measurement, but the information must first come from clinicians. The more signals from physicians, the higher the possibility in avoiding immunochemistry errors in the future. In order to achieve this, the physician taking care of the geriatric population should be familiar with the limitations of immunochemistry.

## Abbreviations

CRP: C-reactive protein
FT4: Free thyroxine
IGF: Insulin-like growth factor
PTH: Parathyroid hormone
RFs: Rheumatoid factors
TBG: Thyroxine-binding globulin
TSH: Thyroid-stimulating hormone.
